# Metal Ions Supported Porous Coatings by Using AC Plasma Electrolytic Oxidation Processing

**DOI:** 10.3390/ma13173838

**Published:** 2020-08-31

**Authors:** Krzysztof Rokosz, Tadeusz Hryniewicz, Steinar Raaen, Sofia Gaiaschi, Patrick Chapon, Dalibor Matýsek, Kornel Pietrzak, Monika Szymańska, Łukasz Dudek

**Affiliations:** 1 Faculty of Mechanical Engineering, Koszalin University of Technology, Racławicka 15–17, PL 75-620 Koszalin, Poland; Tadeusz.Hryniewicz@tu.koszalin.pl (T.H.); kornel.pietrzak@s.tu.koszalin.pl (K.P.); monika.szymanska@s.tu.koszalin.pl (M.S.); 2 Department of Physics, Norwegian University of Science and Technology (NTNU), Realfagbygget E3-124 Høgskoleringen 5, NO 7491 Trondheim, Norway; steinar.raaen@ntnu.no; 3 HORIBA FRANCE S.A.S., Boulevard Th Gobert-Passage Jobin Yvon, CS 45002-91120 Palaiseau, France; sofia.gaiaschi@horiba.com (S.G.); patrick.chapon@horiba.com (P.C.); 4 Institute of Geological Engineering, Faculty of Mining and Geology, VŠB—Technical University of Ostrava, 708-33 Ostrava, Czech Republic; dalibor.matysek@vsb.cz

**Keywords:** plasma electrolytic oxidation (PEO), micro arc oxidation (MAO), titanium, calcium, magnesium, zinc, copper

## Abstract

Coatings enriched with zinc and copper as well as calcium or magnesium, fabricated on titanium substrate by Plasma Electrolytic Oxidation (PEO) under AC conditions (two cathodic voltages, i.e., −35 or −135 V, and anodic voltage of +400 V), were investigated. In all experiments, the electrolytes were based on concentrated orthophosphoric acid (85 wt%) and zinc, copper, calcium and/or magnesium nitrates. It was found that the introduced calcium and magnesium were in the ranges 5.0–5.4 at% and 5.6–6.5 at%, respectively, while the zinc and copper amounts were in the range of 0.3–0.6 at%. Additionally, it was noted that the metals of the block S (Ca and Mg) could be incorporated into the structure about 13 times more than metals of the transition group (Zn and Cu). The incorporated metals (from the electrolyte) into the top-layer of PEO phosphate coatings were on their first (Cu^+^) or second (Cu^2+^, Ca^2+^ and Mg^2+^) oxidation states. The crystalline phases (TiO and Ti_3_O) were detected only in coatings fabricated at cathodic voltage of −135 V. It has also been pointed that fabricated porous calcium–phosphate coatings enriched with biocompatible magnesium as well as with antibacterial zinc and copper are dedicated mainly to medical applications. However, their use for other applications (e.g., catalysis and photocatalysis) after additional functionalizations is not excluded.

## 1. Introduction

Titanium and its alloys, thanks to their properties, have found wide range of applications, e.g., in the aviation industry (fittings, screws, hull frames, brake assemblies, hydraulic hoses, shovels, shafts, afterburner covers and hot air ducts), automotive industry (valve and suspension assemblies, pulleys, steering gears, connecting rods, drive shafts, springs and crankshafts), architecture (claddings and roofing, concrete reinforcement and renovation of monuments), chemical industry (tanks, pumps, pressure reactors, pipes and pipelines), maritime industry (deep-water hulls, submarines, underwater ball valves, data recording devices, parts of lifeboats and anodes for cathodic protection) and medicine (surgical tools, endodontic files and implants) [1]. Electrochemical treatments, such as electropolishing [2], with high current density [3] or high voltage [4], as well as with acceleration of magnetic field [5], are used to create the passive nanolayers. On the other hand, the plasma electrolytic oxidation (PEO) processes [6] are used to fabricate the micro-porous coatings on titanium [7] and its alloys, such as Ti6Al4V [8], NiTi [9], Ti-15Mo [10] and Ti-Nb-Zr [11], most often under DC [12] and AC [13] conditions. It was also found that, in the PEO process performed in water electrolytes, a smaller increase in current value is associated with the formation of a compact oxide film on the anode surface, constituting a barrier layer. The initially formed oxide layer is dissolved, that is associated with the observed increase in current value. This can be referred directly to the voltage value, at which the material melting/pulping in that electrolyte may be observed. Further increase of PEO voltage is responsible for the process of anode surface re-passivation, with the creation of porous oxide structure. The electric field strength in the oxide coating reaches its critical value, above which it is punctured by the collision or tunnel ionization, which is accompanied by small electrical discharges on the entire surface. In addition to the phenomenon of collision ionization, the phenomenon of thermal ionization and a development of larger arcing are observed. At the next voltage stage, there is a partial occluding of thermal ionization, by cumulating negative charge in the creating of compact and thick oxide layer. Further polarization of surface resulted in arcing, penetrating the coating down to the substrate, which is responsible for the destruction of earlier created compact coating [14,15].

The authors’ studies, carried out on coatings fabricated on titanium in the electrolyte based on concentrated phosphoric acid (H_3_PO_4_) with Cu(NO_3_)_2_∙3H_2_O, indicate clearly that, up to the DC voltage of about 100 V, the occurring process may be recognized as a standard anodization, characterized by visible current peaks symbolizing material surface dissolution as well as its secondary re-passivation. After a critical voltage is crossed, a puncture of the passive layer is noticed with the effect of current peak and plasma inflammation in the electrolyte. Under further increasing of the DC voltage, a stabilization of conditions in the generated plasma is observed. It is interesting that, during the process, with the luminescence phenomenon occurring, the treated metal does not have to be coated with an expected layer. It was found that, for the electrolyte containing copper (II) nitrate (V), the lowest voltage at which the porous coating may be formed is about 450 V [16], whereas, for the electrolyte with magnesium nitrate (V), this minimum voltage is about 500 V [17]. One should note that increasing the voltage of PEO treatment results in the growth of current density during the oxidation. Moreover, it was noticed that, in the case of coatings enriched with copper, by increasing the PEO voltage of 200 V, about triple growth of thickness appears, whereas fivefold increase of salt concentration in the solution results in the threefold growth of their thickness. It was also found that the passivation current for the same corrosion potential rises along with increasing of the PEO voltage and decreases along with the growth of the amount of salt in the electrolyte, which may prove of the corrosion resistance of the fabricated coatings. It should be noted that fabrication of coatings of the same electrochemical properties is possible in electrolytes of lesser amount of salt in the solution and lower PEO voltage. Several years of research have shown that it is possible to obtain in DC-PEO process the calcium–phosphate coatings (hydroxyapatite-like structures) [18], which can be enriched with magnesium ions [19], resulting in faster wound healing, as well as antibacterial zinc [20] and copper [21] ions. In addition, it was found that the cerium-doped hydroxyapatite coatings have antimicrobial properties [22]. Although the main research on PEO coatings is focused on biomaterial application, due to their unique porosity and enrichment with selected biocompatible elements, they can also be used in other areas of industry due to their unique porosity.

Thus, the coatings fabricated in the processes of plasma electrolytic oxidation (PEO) have an expanded surface stereometry, which can be used both, for the production of different types of catalysts [23,24], as well as the biocompatible coatings [25,26]. It is important that, in a process that lasts several to tens of minutes, it is possible to produce a coating with the desired porosity and chemical composition. Moreover, further modification of the received surfaces is possible by thermal, hydrothermal treatments, or re-oxidation by PEO in other solution. An example of a catalyst for carbon oxidation reaction of soot, used in the cars with Diesel engine, is the coating by PEO process [27,28]. Other application of PEO coatings is the use of their oxide surfaces as catalysts in biomass gasification process. The example is a PEO surface as a catalyst that allows to obtain over 80% of naphthalene decomposition at the temperature of 1123 K [29]. Another way to functionalize the PEO surfaces fabricated may be their use as catalysts for desulfurization of heavy fractions emerging in the process of petroleum refining. Catalysts used in the conversion of CO to CO_2_, occurring under high temperature conditions (373–773 K), may also be fabricated by PEO. In addition, it was noted that the surfaces containing Cu and Co were characterized by the highest catalytic activity, whereas those enriched with Cu and Fe, the least one [30]. Photocatalytic properties exhibit PEO coatings, consisting of titanium (IV) oxides (TiO_2_), occurring predominantly in the system as an anatase or rutile [31]. Both phases are characterized by the presence of a prohibited/forbidden band, with the energy difference between the valence electronic shell and the conduction band equaling 3.0 eV for rutile and 3.2 eV for anatase. It is considered that the anatase, due to its band structure, wide forbidden band and a low-lying valence electronic shell under the influence of UV radiation in the aquatic solution, has photocatalytic properties, which are related to the formation of hydroxyl radicals [32,33]. It should be mentioned that the phase transformation of anatase to rutile occurs most often as a result of high temperature processes. Annealing/heating of the PEO coating, containing only anatase in its structure at 973 K for 1 h, may result in the transformation of about 25% anatase to rutile, while using a temperature of 1173 K causes the transformation of about 86% anatase [34]. A similar phase transformation was observed in the PEO coatings obtained with the DC voltages in the range of 250–550 V in aqueous solution of sodium phosphate(V). It was also found that the use of additional stage of functionalization of the PEO coating through a 20-h exposure in baths containing selected cations of transitional metals (Ti^4+^, Cr^3+,^ Zn^2+^, Ni^2+^, Co^3+^, Fe^3+^ and Cu^2+^) or rare earth metals (Dy^3+^, Nd^3+^, La^3+^ and Ce^4+^), in each case, improved the photocatalytic properties of the PEO surfaces [35].

Fabricating biocompatible surfaces by the PEO treatment is possible at different DC and AC voltages in aqueous solutions, mostly based on Ca(CH_3_COO)_2_·H_2_O with different additions, e.g., C_3_H_7_CaO6P [36], Na_5_P_3_O_10_ [37] and (NaPO_3_)_6_ [38]. It should also be noted that additions such as magnesium results in a faster wound healing, strontium stimulates bone growth and the additions of silver, zinc and copper cause an increase in antibacterial activity of fabricated coatings [39]. To increase coating biocompatibility additionally, the hydroxyapatite is introduced, using the phenomenon of electrophoresis [40].

It should also be pointed out that it is possible to fabricate the hard coatings using the PEO method by, for example, adding to the electrolytes of the nanopowders of Si_3_N_4_ [41], or Al_2_O_3_ [42]. It was also found, in tribological studies, that the content increase of nanoparticle results in the increase of the wear resistance of these coatings. Furthermore, the addition of cerium nanoparticles (50 nm) to the electrolyte increases surface corrosion resistance and the wear resistance [43]. It was noticed that the received PEO surfaces may have dry lubricant properties, and thereby reduce the coefficient of friction [44]. Information can also be found about that the proper selection of electrolyte composition and parameters of the PEO processes gives the possibility of obtaining coatings with a low abrasion [45], resistant to wear [46], of required hardness [47] and low coefficient of friction [48]. On the other hand, reduction of the friction coefficient of the obtained PEO coatings may be achieved using, e.g., the duplex technique, combining the PEO treatment with the ion implantation in plasma [49], by introducing into electrolyte the 150–550-nm polytetrafluoroethylene (PTFE) molecules and 150-nm carbon fibers [50] or by applying a layer of graphite as a solid lubricant [51]. It is also worth mentioning that the PEO coatings may be used to manufacture gas (H_2_ and CO) and humidity sensors [52,53], as well as sensors for the electromagnetic radiation protection [54].

Despite of the fact that the high potential of technology for fabricating porous coatings by DC Plasma Electrolytic Oxidation (DC-PEO) techniques, which we presented previously, there is still a hunger for the questions that have arisen during the research regarding AC-PEO processing at kilohertz frequencies. In this paper, we attempt to answer the questions whether it is possible to obtain coatings with chemical composition, corrosion resistance and thickness in the AC-PEO process different than those ones produced by the DC-PEO method. In the case of biomaterials, it is important to find coatings with different porosities, with a composition similar to hydroxyapatite enriched with bactericidal elements and those that can accelerate wound healing. Reducing the thickness of the coatings is also very important, as it may affect the faster growth of bone tissue into it. It appears that, with the use of that technique, it is possible in a few-minute treatment—with a “simple” setup relative to CVD (Chemical Vapor Deposition) or PVD (Physical Vapor Deposition)—to fabricate the porous coatings enriched with selected chemical elements at the same time with the desired surface roughness/porosity. As mentioned above, the presented biological studies showed what kind of coatings can be applicable as biomaterials.

## 2. Materials and Methods

### 2.1. Materials and Sample Preparation

**Figure 1:**
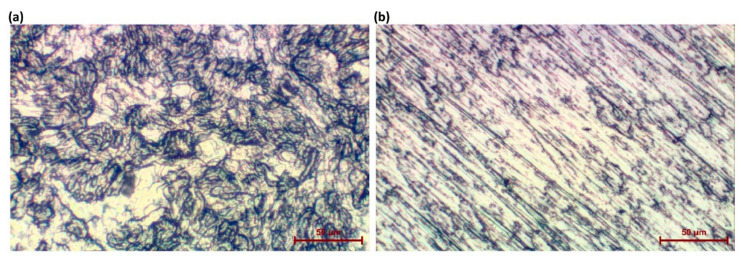
The optical images of samples: (**a**) as received; and (**b**) mechanically grounded.

For all experiments, the titanium samples of *Titanium Grade 2* delivered by Bimo Tech Co, Wrocław, Poland, with the composition shown in Table 1, were used. The delivered material was cut with water jet to obtain a size of 10 mm × 10 mm × 2 mm, and a titanium wire (ø1 × 70 mm) was welded to each sample to obtain an electrical connection in the PEO process. The surface of the sample was ground with SiC sandpaper with the gradation of #1200, followed by ultrasonic cleaning in acetone and drying in a stream of air (Figure 1). The PEO process setup consisted of an AC power supply (ASTERION POWER SOURCE/AMETEK, Berwyn, PA, USA) with a negative clamp connected to the cathode, which is made of austenitic AISI 316L (Table 2) stainless steel, while a positive clamp was connected to the treated sample, i.e., titanium (2.8 cm^2^). The titanium samples were treated in different electrolytes by AC-PEO (5 kHz square waveform), with abbreviations shown in Table 3.

### 2.2. Characterization Methods

The equipment and software with their producers, related to three measuring methods, are listed in Table 4. In detail, the parameters of those methods are presented below. An optical microscope, Trinocular Metallographic Microscope OPMT NJF-120A (OPMT NJF-120A/Nanjing Jiangnan Novel Optics Co., Ltd., Nanjing, China), a scanning electron microscope, Quanta 250 FEI with Low Vacuum and ESEM mode (FEI Quanta 650/Field Electron and Iron Company, Hillsboro, OR, USA), a field emission cathode, and an energy dispersive EDS system in a Noran System Six (EDAX Apollo X/EDAX AMETEK, Jersey City, NJ, USA) with nitrogen-free silicon drift detector were used. X-ray photoelectron spectroscopy (XPS) measurements on the studied sample surfaces were performed by means of SCIENCE SES 2002 instrument (SCIENCE SES 2002/ScientaOmicron, Uppsala, Sweden) using a monochromatic (Gammadata-Scienta, ScientaOmicron, Uppsala, Sweden) Al K(alpha) (*hυ* = 1486.6 eV) X-ray source (18.7 mA, 13.02 kV). Scan analyses were carried out with an analysis area of 1 mm × 3 mm and a pass energy of 500 eV, with the energy step 0.2 eV and a step time 200 ms. The binding energy of the spectrometer was calibrated by the position of the Fermi level on a clean metallic sample. The power supplies were stable and of high accuracy. The experiments were carried out in an ultra-high vacuum system with a base pressure of about 6 × 10^−8^ Pa. All the binding energy values presented in this paper were charge corrected to C 1s at 284.8 eV. Glow discharge optical emission spectroscopy (GDOES) measurements on the PEO oxidized titanium samples were performed using a Horiba Scientific GD Profiler 2 instrument (GD Profiler 2/HORIBA Scientific, Palaiseau, France). A radio frequency (RF) pulsed source was used to generate plasma under the following conditions: pressure, 700 Pa; power, 40 W; frequency, 3000 Hz; duty cycle, 0.25; and anode diameter, 4 mm. The structure was studied by Powder X-ray diffraction (XRD) using a Bruker-AXS D8 Advance instrument (BRUKER Corporation, Billerica, MA, USA), with the 2Ѳ/Ѳ geometry using a LynxEye position-sensitive detector (radiation CuK/Ni filter, 40 kV, 40 mA, 0.014 2 Ѳ and interval of 0.25 s (BRUKER Corporation, Billerica, MA, USA). The corrosion potentiodynamic polarization tests by using Atlas 98 EII (Atlas-Sollich, Rebiechowo, Poland) were performed in a Ringer’s solution after 24-h immersion in electrolyte. The setup consisted of a three-electrode system, using a platinum counter electrode and reference electrode—saturated calomel electrode (SCE)—with a step of 1 mV/s. For estimation of the corrosion resistance, passive current values at the anodic polarization of +1200 mV vs. SCE, were taken. To determine whether two dependent samples were selected from the populations, having the same distribution, i.e., to show significant differences between the samples fabricated at different cathodic voltages, the non-parametric statistical Wilcoxon signed-rank test at the 5% significance level was used. For data processing, Casa XPS 2.3.14 (Casa Software Ltd., Teignmouth, Devon, UK) and Matlab 2017a (MathWorks, Inc., Natick, MA, USA) were used.

## 3. Results and Discussion

**Figure 2:**
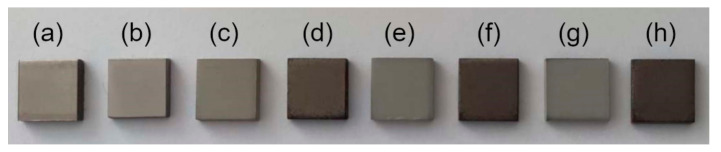
The photo of samples: (**a**) as received and after waterjet cutting; (**b**) after mechanical treatment with SiC sandpaper with a gradation of #1200 and after PEO: (**c**) TiZnCu-35; (**d**) TiZnCu-135; (**e**) TiZnCuCa-35; (**f**) TiZnCuCa-135; (**g**) TiZnCuMg-35; and (**h**) TiZnCuMg-135.

**Figure 3:**
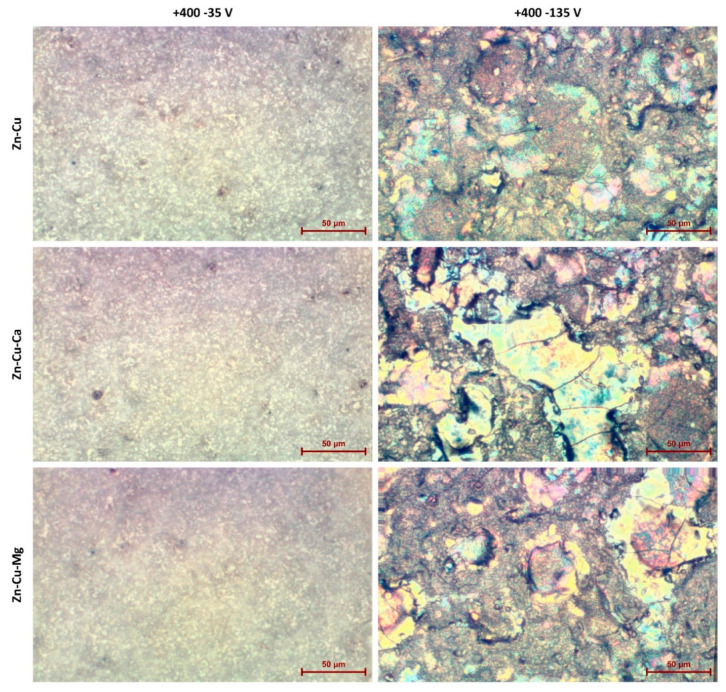
The optical images of AC-PEO coatings fabricated at negative pulses −35 V and −135 V with the same positive voltage of +400 V.

**Figure 4:**
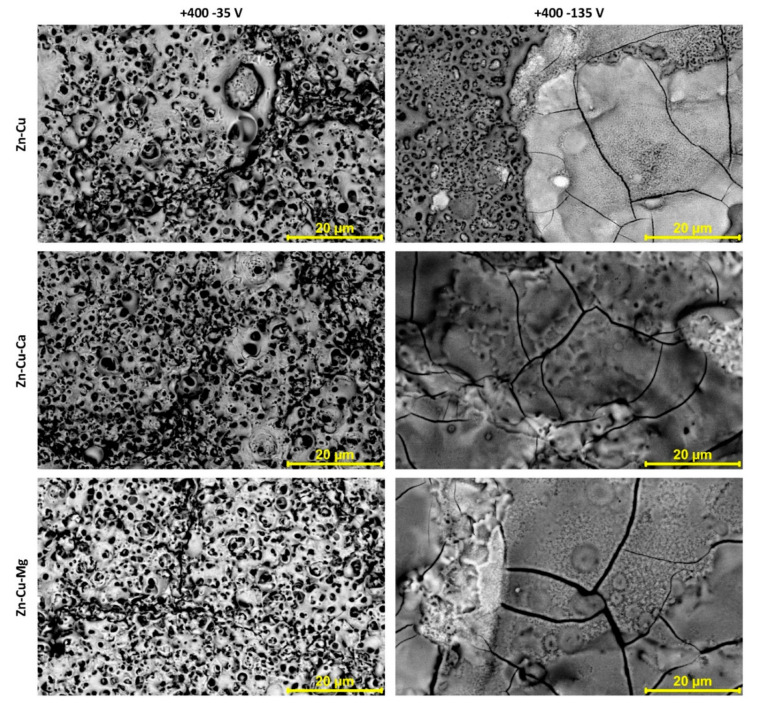
The SEM images of AC-PEO coatings fabricated at negative pulses −35 V and −135 V with the same positive voltage of +400 V.

The scanning electron microscopy images, with magnification of 4000× show that there are significant differences between the coatings fabricated under different cathodic conditions (−35 V and −135 V) and the same anodic voltage of +400 V, as presented in Figure 2. Those samples obtained under +400/−35 V have a porosity similar to those obtained under DC conditions [17,18,19]. The coatings fabricated at −35 V cathodic voltage revealed less cracked surfaces than those obtained at −135 V. The usage of a cathodic voltage of −35 V provides coatings with a “more uniform” porous structure, but still with the observed regions that differ regarding pores size and shape (Figure 3 and Figure 4). It should also be noted that the biggest cracks were observed for the coatings obtained in electrolytes containing magnesium ions (TiZnCuMg-35 and TiZnCuMg-135). Increasing the cathodic voltage causes the supply of much more energy, which results in melting the coating and appearance of distinctive artifacts with visible cracks. The samples produced at a higher negative cathodic voltage have characteristic discoloration that indicates a very high temperature at the surface of the sample during the process (Figure 3). It should be mentioned that cracks that do not cause the delamination of the coating in the case of biomedical applications in implantology, thus they do not have a negative effect on the cells. It is important that, during the AC-PEO treatment, half of the time the sample is an anode. It follows that the sample is anode for a half of the period, i.e., is treated in the PEO process, and in the second period is a cathode, which is an electroplating-like process. Thus, it can be expected that, during the “polarity reversal period”, the reduction reactions of, e.g., Cu^2+^ to Cu^+^ and/or Ti^4+^ to Ti^3+^, occurs, and it can be noticed that an outer coating is oxidized when the sample becomes an anode again.

The examples of EDS spectra with their analyses of the obtained coatings are presented in Figure S1 and the averages with the standard deviations of all obtained EDS results are presented in Table 4. The amount of phosphorus in coatings fabricated during the PEO process is in the range of 2.82 ± 0.15 to 3.68 ± 0.23 at%, for the coatings obtained at a cathodic voltage of −135 V, while, at −35 V, the concentration is in the range of 12.05 ± 0.08 to 12.37 ± 0.2 at% (Table S1). This proves that, at −35 V cathode voltage, we mainly have phosphates, while, at −135 V, we have oxides and/or hydroxides. In the case of zinc and copper, they were not detected in the coatings produced at −135 V, and for −35 V their values were lower than 1 at%. Calcium and zinc were present only in the coatings obtained for the −35 V cathode voltage and were equal to 0.47 ± 0.02 and 0.62 ± 0.05 at%., respectively.

**Figure 5:**
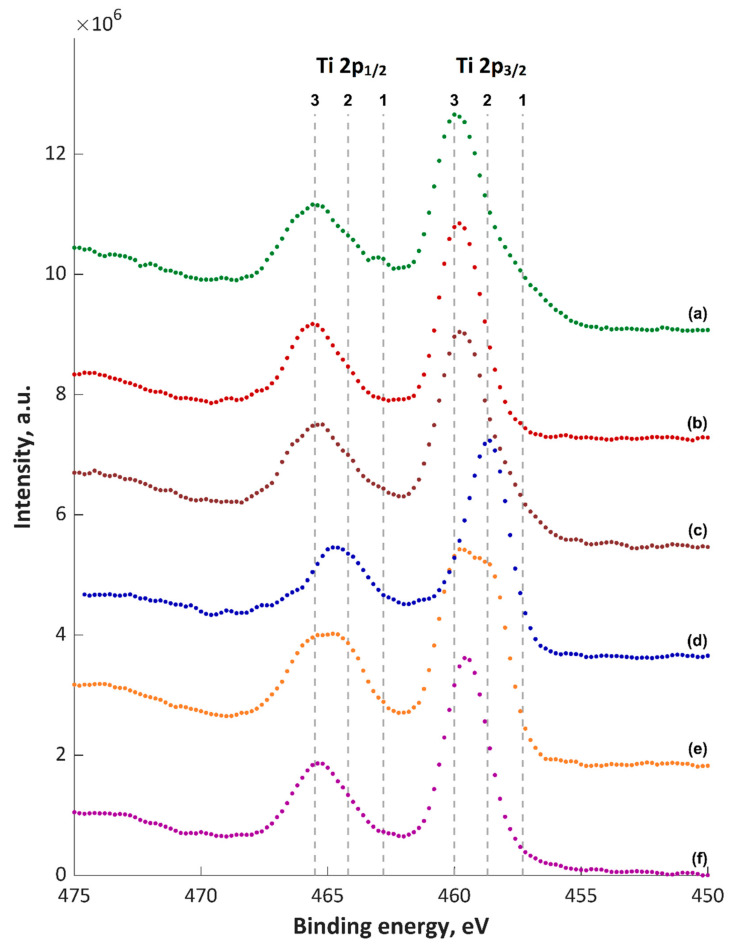
XPS Ti2p spectra of PEO coatings: (**a**) TiZnCu-35; (**b**) TiZnCu-135; (**c**) TiZnCuCa-35; (**d**) TiZnCuCa-135; (**e**) TiZnCuMg-35; and (**f**) TiZnCuMg-135; 1, Ti_2_O_3_; 2, 3, TiO_2_, CaTiO_3_ and/or Ti_3_(PO_4_)_4_.

**Figure 6:**
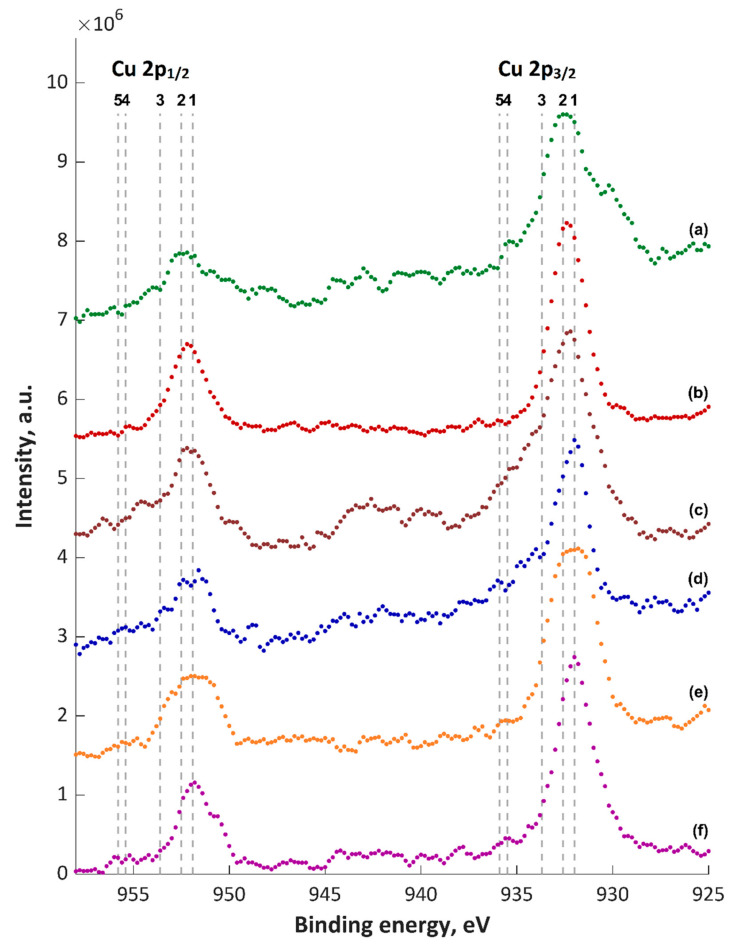
XPS Ti 2p spectra of PEO coatings: (**a**) TiZnCu-35; (**b**) TiZnCu-135; (**c**) TiZnCuCa-35; (**d**) TiZnCuCa-135; (**e**) TiZnCuMg-35; and (**f**) TiZnCuMg-135. 1, Cu_2_O; 2, CuO; 3, Cu(OH)_2_; 4, Cu(NO_3_)_2_; 5, Cu_3_(PO_4_)_2_.

**Figure 7:**
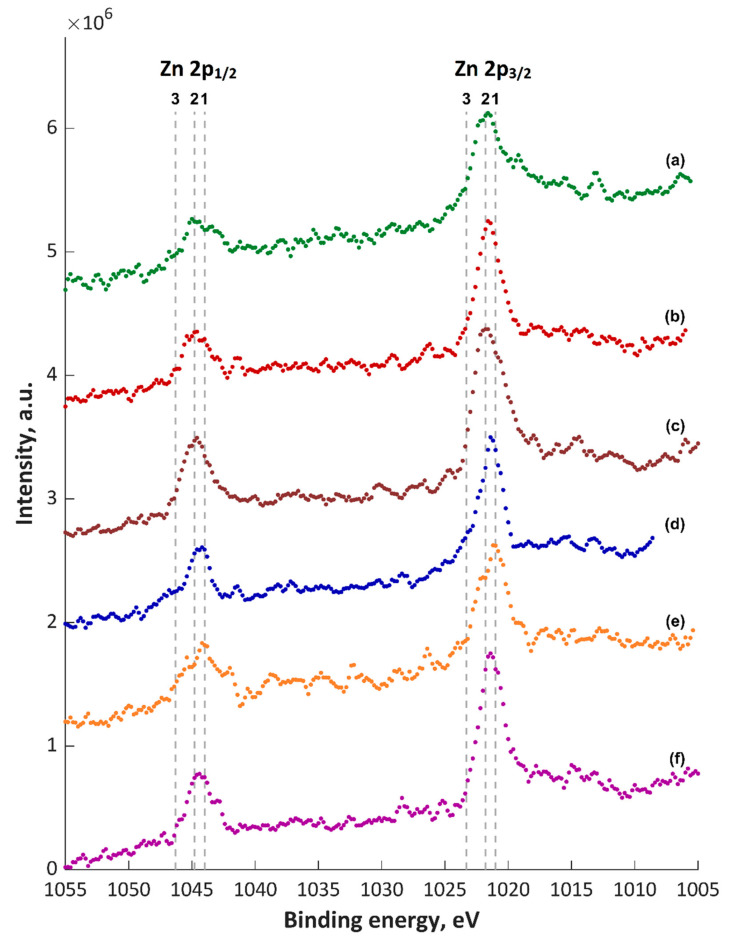
XPS Zn 2p spectra of PEO coatings: (**a**) TiZnCu-35; (**b**) TiZnCu-135; (**c**) TiZnCuCa-35; (**d**) TiZnCuCa-135; (**e**) TiZnCuMg-35; and (**f**) TiZnCuMg-135. 1, ZnO; 2, Zn(OH)_2_; 3, Zn_3_(PO_4_)_2_.

**Figure 8:**
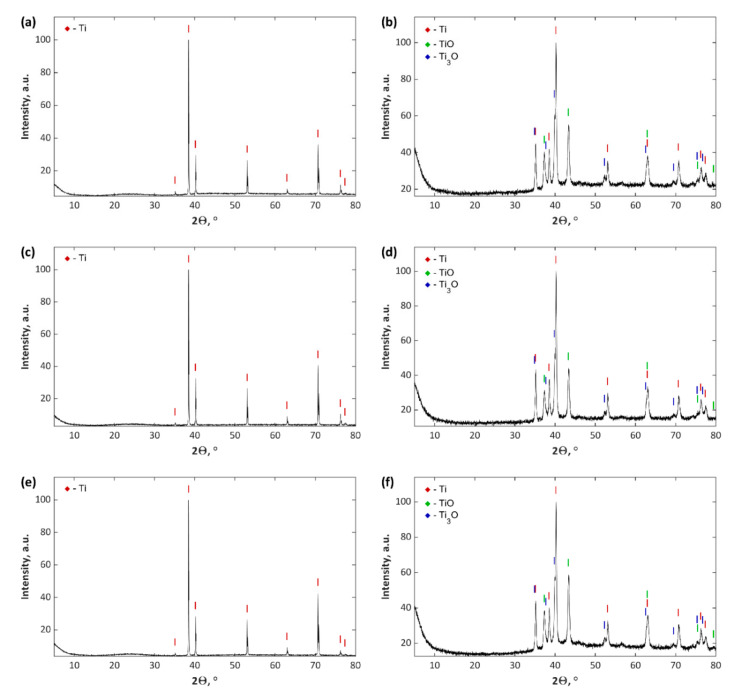
XRD diffractograms of PEO coatings: (**a**) TiZnCu-35; (**b**) TiZnCu-135; (**c**) TiZnCuCa-35; (**d**) TiZnCuCa-135; (**e**) TiZnCuMg-35; and (**f**) TiZnCuMg-135.

**Figure 9:**
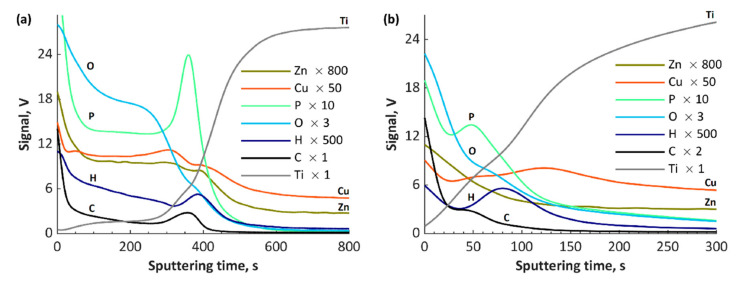
GDOES depth profiles of the coatings: (**a**) TiZnCu-35; and (**b**) TiZnCu-135.

The obtained XPS spectra of top about 5–10-nm layer (5 nm for TiO_2_) are shown in Figure 5, Figure 6, Figure 7, Figure 8 and Figure 9 and Figures S2–S7, and their quantitative analyses are presented in Figures S8–S10 and Table S1. Based on the XPS data of TiZnCu-35 and TiZnCu-135 coatings, it was found that the copper and zinc amounts were equal to 0.4 at% for both coatings. The detected titanium, phosphorus, nitrogen and oxygen amounts were in the range from 3.6 at% (TiZnCu-35) to 4.9 at% (TiZnCu-135), from 27.5 at% (TiZnCu-35) to 27.8 at% (TiZnCu-135), from 2.1 at% (TiZnCu-135) to 2.9 at% (TiZnCu-35) and from 64.4 at% (TiZnCu-135) to 65.2 at% (TiZnCu-35), respectively. Herewith, the ratios Cu/P and Zn/P are equal to approximately of 0.01 for both samples. Additionally, the calculated Ti/P atomic ratios were equal to 0.13 and 0.18 for TiZnCu-35 and TiZnCu-135, respectively.

Based on the analysis of XPS data for the Zn/Cu/Ca-enriched coatings, it was found that the amounts of copper and zinc in TiZnCuCa-35 were equal to 0.6 at% while the amount of calcium was equal to 5.0 at%. Similar results were recorded for TiZnCuCa-135, while the amounts of copper, zinc and calcium were equal to 0.4, 0.5 and 5.4 at%, respectively. The detected titanium, phosphorus, nitrogen and oxygen amounts were in the ranges from 4.5 at% (TiZnCuCa-135) to 4.7 at% (TiZnCuCa-35), from 23.7 at% (TiZnCuCa-135) to 25.5 at% (TiZnCuCa-35), from 1.1 at% (TiZnCuCa-35) to 1.7 at% (TiZnCuCa-135) and from 62.5 at% (TiZnCuCa-35) to 63.8 at% (TiZnCuCa-135), respectively. The atomic ratios of Cu/P and Zn/P are approximately the same and equal to 0.02 for both samples (TiZnCuCa-35 and TiZnCuCa-135), while Ca/P ratios are equal to 0.20 and 0.23, respectively. Additionally, the calculated Ti/P atomic ratios for TiZnCuCa-35 and TiZnCuCa-135 were equal to 0.18 and 0.19, respectively.

Based on the analysis of XPS data for the Zn/Cu/Mg-enriched coatings, it was found that copper, zinc and magnesium amounts in TiZnCuCa-35 sample were equal to 0.3, 0.4 and 5.6 at%, respectively. Analogous results were recorded for TiZnCuCa-135 sample, where the amounts of copper, zinc and magnesium equaled 0.4, 0.4 and 6.5 at%, respectively. The detected titanium, phosphorus, nitrogen and oxygen amounts were in the ranges from 4.7 at% (TiZnCuMg-35) to 6.1 at% (TiZnCuMg-135), from 25.0 at% (TiZnCuMg-135) to 26.3 at% (TiZnCuMg-35), from 1.7 at% (TiZnCuMg-135) to 2.0 at% (TiZnCuMg-35) and from 59.9 at% (TiZnCuMg-135) to 60.7 at% (TiZnCuMg-35), respectively. The calculated atomic ratios of Cu/P, Zn/P and Mg/P were equal to 0.01, 0.02 and 0.21 for TiZnCuMg-35 sample, respectively. On the other hand, the calculated Cu/P and Zn/P atomic ratios were the same and equal to 0.02, while the Mg/P ratio was equal to 0.26 for TiZnCuMg-135 sample. Additionally, the calculated Ti/P atomic ratios for TiZnCuMg-35 and TiZnCuMg-135 were equal to 0.18 and 0.24, respectively.

The maxima of binding energies (BE, eV) of PEO coatings are presented in Table S2, and they were analyzed with regard to the literature data collected in Table S3. The maxima of the main peak of Cu 2p_3/2_ spectra were equal to 932.6 and 932.4 eV, which can be interpreted as the first oxidation state (Cu^+^). It was also noted that the strongest signals corresponding to Cu^2+^ satellites were recorded mainly for TiZnCu-35 and TiZnCuCa-35 samples, which was not so visible for the samples treated at −135 V sample, most likely related to a reduction during the period in which the sample is the cathode. The binding energies, equal to 1021.2–1021.8 eV (Zn 2p_3/2_) and 459.8–460.0 eV (Ti 2p), suggest mainly the presence of Zn^2+^ and Ti^4+^ in the top layer. Analyses of P 2p (134.0 and 133.8 eV) and O 1s (531.6 and 531.4 eV) reveal the presence of phosphates or metaphosphates. In addition, it should be noted that, for the samples TiZnCuCa-135, BE of O 1s is equal to 530.6 eV, which suggests the large amount of oxides that can origin from CaO (529.4–531.3 eV), MgO (530.0–532.1 eV), Cu_2_O (530.5 eV) and/or TiO_2_ (529.9 eV). The rest of binding energies in the range of 531.4–531.6 eV can be classified as hydroxides and salts (phosphates and nitrites), and their mix. It has to be pointed out that the energy of 531.6 eV in the O 1s spectrum can refer to the adsorbed oxygen. The binding energies in the range from 399.8 to 402.0 eV in N 1s spectra give information about a possible occurrence of nitrogen, as inorganic compounds, as well as possible organic contaminations. Based on Ti 2p_3/2_ spectra (Figure 5), it can be seen that most likely in top surface of PEO coatings which include mostly TiO_2_, CaTiO_3_ and/or Ti_3_(PO_4_)_4_ may be indicated by Points 2 (BE = 458.7 eV) and 3 (BE = 460.0 eV), and small amount pf Ti_2_O_3_ (Point 1; BE = 457.3 eV). In case of Cu 2p_3/2_ spectra (Figure 6) it can be presumed that the top of fabricated coatings containing mainly two oxides Cu_2_O (Point 1; BE = 932 eV) and CuO (Point 2; BE = 932.6 eV) as well as hydroxide of copper (Point 3; BE = 933.7 eV). Due to the electrolyte used, the possibility of the of nitrate Cu(NO_3_)_2_ and phosphate Cu_3_(PO_4_)_2_ formation should also be assumed, however in smaller amount. Therefore, two lines are marked in Figure 6 with Items 4 (BE = 935.5 eV) and 5 (935.9 eV), respectively. In the case of zinc Zn 2p_3/2_, spectra analysis shows that the top of the PEO coatings contain mainly oxide ZnO (Point 1; BE = 1021 eV) and hydroxide Zn(OH)_2_ (Point 2; BE = 1021.8 eV) of zinc with a smaller amount of phosphate Zn_3_(PO_4_)_2_ (Point 3; BE = 1023.3 eV).

The recorded X-ray diffractograms for the coatings, prepared under the cathodic voltage of −35 V, besides the observed titanium substrate peaks, revealed a halo in the range of 20–30° (Figure 8a,c,e). One may assume that the obtained coatings under these voltage conditions are most likely characterized by the occurrence of only amorphous phase; however, the usage of −135 V cathodic voltage allows obtaining coatings with titanium oxides (TiO and Ti_3_O) in their crystalline phase (Figure 8b,d,f). These results indicate that the crystalline phase contains stoichiometric and non-stoichiometric titanium oxides for coatings produced at −135 V, which fully correlates with the results from EDS. The remaining chemical compounds, which were recorded by the XPS method, are most likely contained in the amorphous phase.

**Figure 10:**
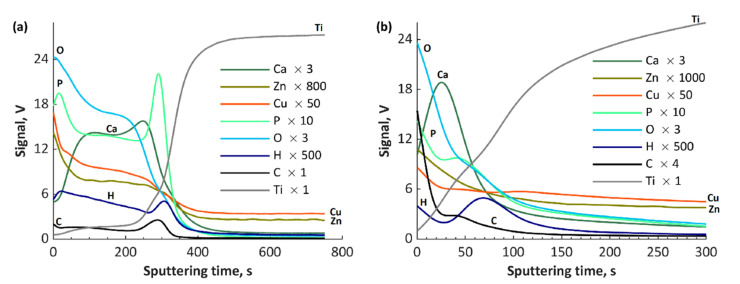
GDOES depth profiles of the coatings: (**a**) TiZnCuCa-35; and (**b**) TiZnCuCa-135.

**Figure 11:**
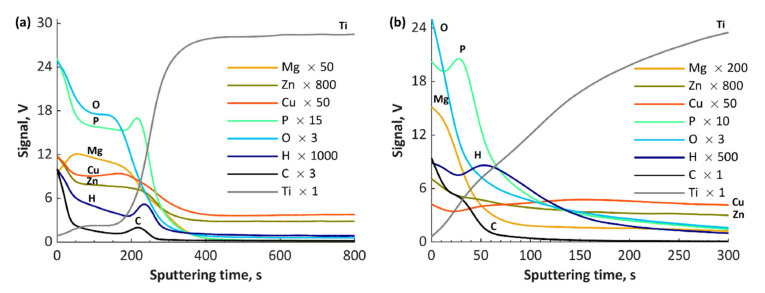
GDOES depth profiles of the coatings: (**a**) TiZnCuMg-35; and (**b**) TiZnCuMg-135.

The GDOES results of fabricated coatings are presented in Figure 9, Figure 10 and Figure 11. The GDOES signals of phosphorus, oxygen, hydrogen and carbon, as well as of copper, zinc, calcium and magnesium, have their maximal values in the first (porous) and second (semiporous) layers, whereas titanium signals in those regions are small. In the third (transition) layer, the titanium signals increase, while the other signals (Ca, Ca, Mg, P, O, H and C), decrease. It is important to note that the coatings’ thicknesses for all the samples obtained with the use of cathodic voltage of −35 V is several times higher (3–4 times) than those fabricated at −135 V. In the case of TiCuZn-135, TiCuZnCa-135 and TiCuZnCa-135 coatings, there are clearly visible enrichments of zinc, calcium and magnesium.

**Figure 12:**
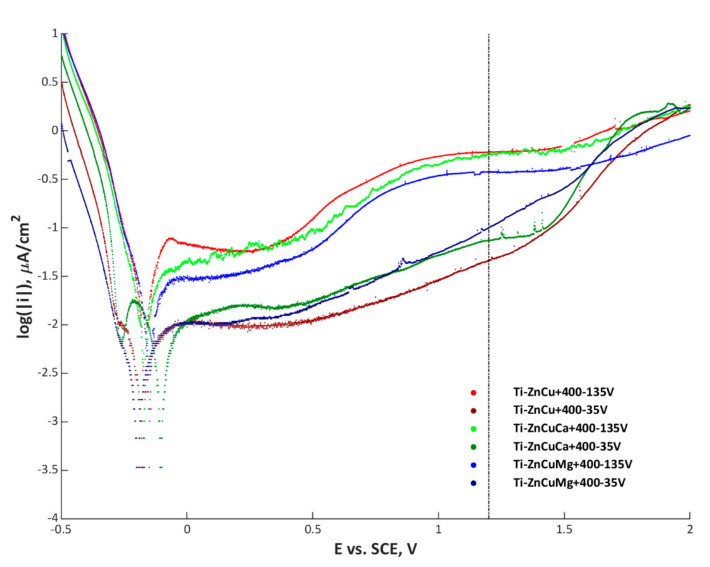
Polarization curves of fabricated coatings.

In Figure 12, the polarization curves of all fabricated coatings are presented. It was found that thicker, compact, passive transition layers were obtained for coatings gained at cathodic voltage of −35 V, compared to the ones fabricated at −135 V. Based on the Wilcoxon rank sum test, for the samples fabricated at both cathodic potentials, i.e., −35 and −135 V, a calculated *p*-value equal to 0.1 indicates the rejection of the null hypothesis of the medians equality at the default 5% significance level. It is important that the corrosion resistance of porous PEO coatings depends mainly on the transition layer, which is located between the porous layer and the substrate (titanium). The more compact is the layer, the better is the corrosion resistance of the entire electrochemical system (substrate–coating–solution). In the case of a high cathodic voltage (−135 V), the thin and porous coating is additionally cracked, which makes it less passive. In the case of coatings obtained at −35 V, the thicker and the more compact they are, the more passive are the systems.

## 4. Conclusions

In this article, we present the possibility of obtaining porous coatings on a titanium substrate in an electrolyte based on the concentrated orthophosphoric acid (85 wt%), containing copper and zinc nitrates, as well as calcium or magnesium nitrates, using the AC-PEO method. It is shown that the use of alternating voltage with a square wave frequency of 5 kHz allows obtaining coatings with a varied surface structure, dependent on the value of the cathode voltage applied. The obtained PEO coatings at the cathode voltages of −35 and −135 V have significantly different corrosion resistances, associated with the surface cracks, and thicknesses of the obtained coatings, as evidenced by the SEM images and the analyses of GDOES signals. Examination of the crystalline phase showed that the use of −135 V voltage results in obtaining TiO and Ti_3_O in the crystalline phase, in contrast to the surfaces obtained at the cathode voltage of −35 V. The XPS tests of the about 5-nm surface layer showed that, regardless of the cathode voltage and type of electrolyte used, the amounts of the implanted zinc and copper were about the same and ranged from 0.3 to 0.6 at%. In addition, it is presented that it is possible to incorporate both magnesium and calcium in larger amounts than zinc and copper, with the amount of magnesium ranging from 5.6 (TiZnCuMg-35) to 6.5 at% (TiZnCuMg-135) and calcium from 5.0 (TiZnCuCa-35) to 5.4 at% (TiZnCuCa-135). Analysis of XPS spectra showed that, in the 10-nm surface layer, besides the impurities, there are in all samples phosphates or diphosphates, Zn^2+^, Ti^4+^ and, in selected samples, Ca^2+^ (TiZnCuCa-35 and TiZnCuCa-135) or Mg^2+^ (TiZnCuMg-35 and TiZnCuMg-135). Analysis of the Cu 2p_3/2_ spectrum showed that copper is mainly in the form of Cu^+^ with a small amount of Cu^2+^, as evidenced by the presence of satellites characteristic of copper in the second oxidation state.

The obtained results and discussion lead to utilitarian conclusions, as follows:

- The AC-PEO process on titanium, performed in electrolytes based on concentrated phosphoric acid with the additions of zinc, copper, calcium and/or magnesium nitrites, using the same anodic voltage of +400 V as well as two cathodic ones, i.e., −35 and −135 V, with a frequency of 5 kHz, results in obtaining coatings with similar chemical composition of top layers, but drastically different morphological properties.
- Increase of negative cathodic voltage in the AC-PEO process from −35 to −135 V results in a significant reduction of coatings thickness and corrosion resistance as well as increase in the proportion of the crystalline-to-amorphous phase in the coatings.
- Atomic concentrations of Ca^2+^ and Mg^2+^ ions (5.0–5.4 at% and 5.6–6.5 at%, respectively) in the top layer values differ by one order of the magnitude in comparison to the atomic concentrations of Cu^+^/Cu^2+^ and Zn^2+^ ions (0.3–0.6 at% and 0.4–0.6 at%, respectively) despite the similar concentrations in the electrolyte.
- During the negative voltage pulse in the AC-PEO process, the reductions of some ions, e.g., Cu^2+^ or Ti^4+^, can occur, which results in their presence in the top layers.

## Figures and Tables

**Table 1:** Chemical composition of Titanium Grade 2 (anode in PEO process).

Content of the Elements, wt%					
N	C	H	Fe	O	Ti
<0.03	<0.10	<0.015	<0.30	<0.25	balance

**Table 2:** Chemical composition of AISI 316L (cathode in PEO process).

Content of the Elements, wt%													
Cr	Ni	Mo	Mg	Si	Cu	V	Co	C	Ti	P	S	N	Fe
17.07	10.26	1.9	1.68	0.64	0.19	0.11	0.04	0.03	0.03	0.02	0.04	0.04	balance

**Table 3:** Abbreviated samples’ names.

Abbreviation	Electrolyte Composition	Voltage
TiZnCu-35	1 dm^3^ H_3_PO_4_ (85 wt%) with 250 g Zn(NO_3_)_2_∙6H_2_O and 250 g Cu(NO_3_)_2_∙3H_2_O	+400/−35 V
TiZnCu-135	1 dm^3^ H_3_PO_4_ (85 wt%) with 250 g Zn(NO_3_)_2_∙6H_2_O and 250 g Cu(NO_3_)_2_∙3H_2_O	+400/−135 V
TiZnCuCa-35	1 dm^3^ H_3_PO_4_ (85 wt%) with 167.67 g Zn(NO_3_)_2_∙6H_2_O and 166.67 g Cu(NO_3_)_2_∙3H_2_O and 166.67 g Ca(NO_3_)_2_∙4H_2_O	+400/−35 V
TiZnCuCa-135	1 dm^3^ H_3_PO_4_ (85 wt%) with 167.67 g Zn(NO_3_)_2_∙6H_2_O and 166.67 g Cu(NO_3_)_2_∙3H_2_O and 166.67 g Ca(NO_3_)_2_∙4H_2_O	+400/−135 V
TiZnCuMg-35	1 dm^3^ H_3_PO_4_ (85 wt%) with 167.67 g Zn(NO_3_)_2_∙6H_2_O and 166.67 g Cu(NO_3_)_2_∙3H_2_O and 166.67 g and 166.67 g Mg(NO_3_)_2_∙6H_2_O	+400/−35 V
TiZnCuMg-135	1 dm^3^ H_3_PO_4_ (85 wt%) with 167.67 g Zn(NO_3_)_2_∙6H_2_O and 166.67 g Cu(NO_3_)_2_∙3H_2_O and 166.67 g and 166.67 g Mg(NO_3_)_2_∙6H_2_O	+400/−135 V

**Table 4:** Results of EDS analysis of PEO coatings, at%.

Samples	Elements						
	O	Ti	P	Zn	Cu	Ca	Mg
	Average ± Standard Deviation, at%						
**TiZnCu-35**	71.48 ± 0.13	15.32 ± 0.22	12.08 ± 0.15	0.71 ± 0.03	0.41 ± 0.03		
**TiZnCu-135**	61.97 ± 1.23	34.35 ± 1.45	3.68 ± 0.23	*	*		
**TiZnCuCa-35**	71.99 ± 0.16	14.63 ± 0.06	12.05 ± 0.08	0.5 ± 0.05	0.36 ± 0.05	0.47 ± 0.02	
**TiZnCuCa-135**	56.11 ± 1.9	41.07 ± 2.03	2.82 ± 0.15	*	*	*	
**TiZnCuMg-35**	71.95 ± 0.3	14.2 ± 0.32	12.37 ± 0.2	0.49 ± 0.03	0.37 ± 0.04		0.62 ± 0.05
**TiZnCuMg-135**	56.51 ± 1.45	40.39 ± 1.51	3.1 ± 0.06	*	*		*

* Below limit of quantitation in the EDS method.

## Supplementary Materials

The following are available online at https://www.mdpi.com/1996-1944/13/17/3838/s1, Figure S1: EDS spectra of coatings obtained at applied voltage of +400|−35 V and +400|−135 V, Table S1: Results of XPS analysis of PEO coatings in atomic percentage, Table S2: Maxima of binding energies (eV) of PEO coatings, Figure S2: XPS spectra of coatings enriched in zinc and copper, obtained at applied voltage of +400|−35 V, Figure S3: XPS spectra of coatings enriched in zinc and copper, obtained at applied voltage of +400|−135 V, Figure S4: XPS spectra of coatings enriched in zinc, copper, and calcium, obtained at voltage of +400|−35 V, Figure S5: XPS spectra of coatings enriched in zinc, copper, and calcium, obtained at voltage of +400|−135 V, Figure S6: XPS spectra of coatings enriched in zinc, copper, and magnesium, obtained at voltage of +400|−35 V, Figure S7: XPS spectra of coatings enriched in zinc, copper, and magnesium, obtained at voltage of +400|−135 V, Figure S8: Elemental composition by XPS of top 10 nm of coatings enriched in copper and zinc, obtained at voltage of: (a) +400|−35 V, (b) +400|−135 V, Figure S9: Elemental compositions by XPS of top 10 nm of coatings enriched in copper, zinc, and calcium, obtained at voltages of: (a) +400|−35 V, (b) +400 | −135 V, Figure S10: Elemental composition by XPS of top 10 nm of coatings enriched in copper, zinc, and magnesium, obtained at voltages of: (a) +400|−35 V, (b) +400|−135 V, Table S3: Binding energies (BE, eV) of selected chemical compounds from available literature.
