# Supplementary material for: Metal Ions Supported Porous Coatings by Using AC Plasma Electrolytic Oxidation Processing
Source: Materials (Basel). 2020 Aug 31;13(17):3838. doi: 10.3390/ma13173838 (PMC7503912; doi:10.3390/ma13173838)
Supplement: Supplementary file 1 [file materials-13-03838-s001.pdf]

materials

MDPI

Article

Supplementary Materials: Metal Ions Supported Porous Coatings by Using AC Plasma Electrolytic Oxidation Processing

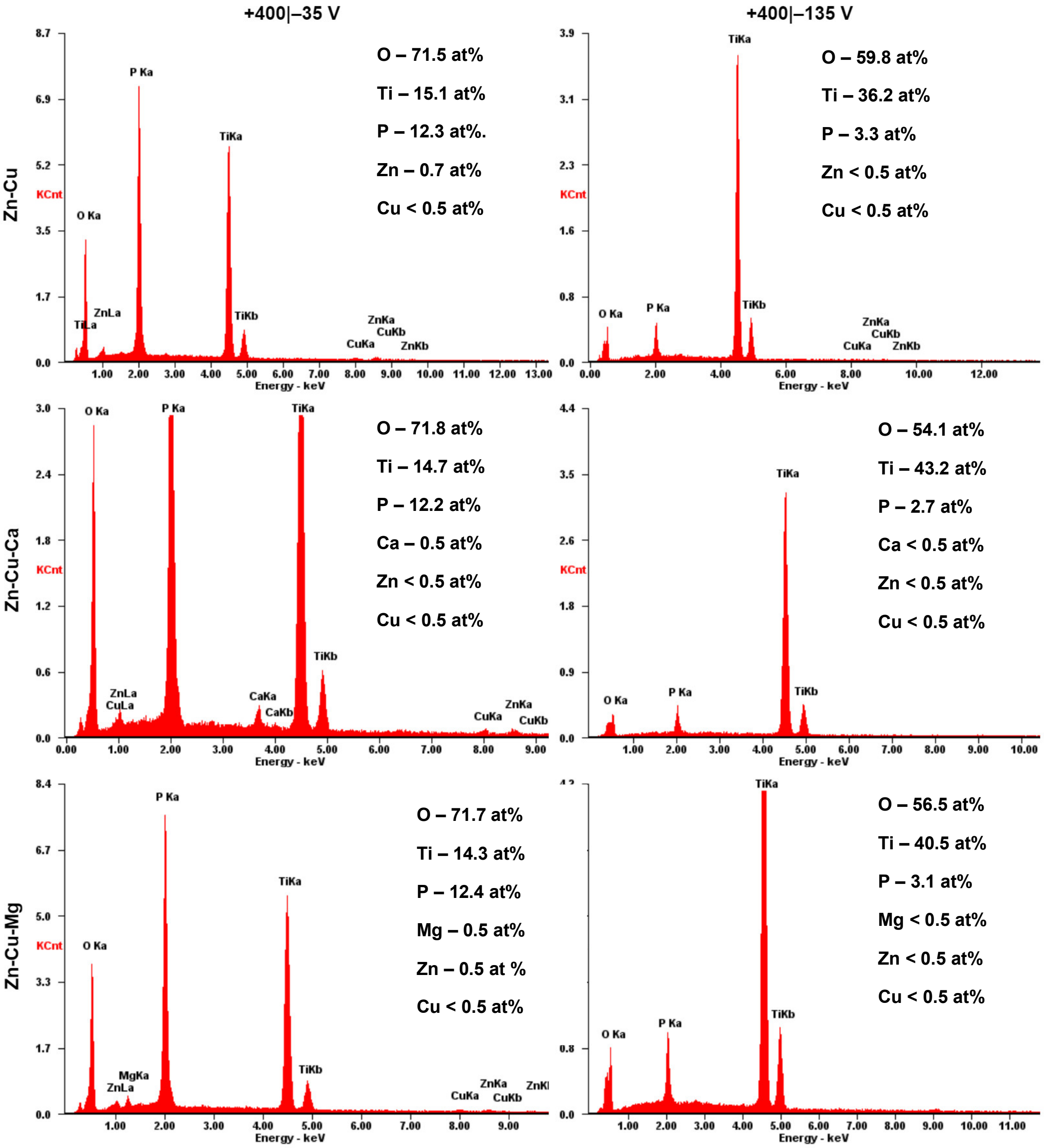

Figure S1. EDS spectra of coatings obtained at applied voltage of +400|–35 V and +400|–135 V.

Table S1. Results of XPS analysis of PEO coatings in atomic percentage.

Sample	Ca	Mg	Zn	Cu	Ti	P	O	N

TiZnCu-35	–	–	0.4	0.4	3.6	27.5	65.2	2.9
TiZnCu-135	–	–	0.4	0.4	4.9	27.8	64.4	2.1
TiZnCuCa-35	5.0	–	0.6	0.6	4.7	25.5	62.5	1.1
TiZnCuCa-135	5.4	–	0.5	0.4	4.5	23.7	63.8	1.7
TiZnCuMg-35	–	5.6	0.4	0.3	4.7	26.3	60.7	2.0
TiZnCuMg-135	–	6.5	0.4	0.4	6.1	25.0	59.9	1.7

Table S2. Maxima of binding energies (eV) of PEO coatings.

Sample	Ca $2p_{3/2}$	Mg 2s	Zn $2p_{3/2}$	Cu $2p_{3/2}$	Ti $2p_{3/2}$	P 2p	O 1s	N 1s
TiZnCu-35	–	–	1021.6	932,6	460.0	134.0	531.6	402.0
TiZnCu-135	–	–	1021.6	932.4	459.8	133.8	531.4	399.8
TiZnCuCa-35	347.6	–	1021.8	932.2	459.8	133.8	531.6	400.6
TiZnCuCa-135	346.8	–	1021.4	932.0	458.6	133.0	530.6	401.0
TiZnCuMg-35	–	89.2	1021.2	931.8	559.8	133.6	531.4	400.4
TiZnCuMg-135	–	89.0	1021.4	932.0	459.6	133.8	531.4	401.6

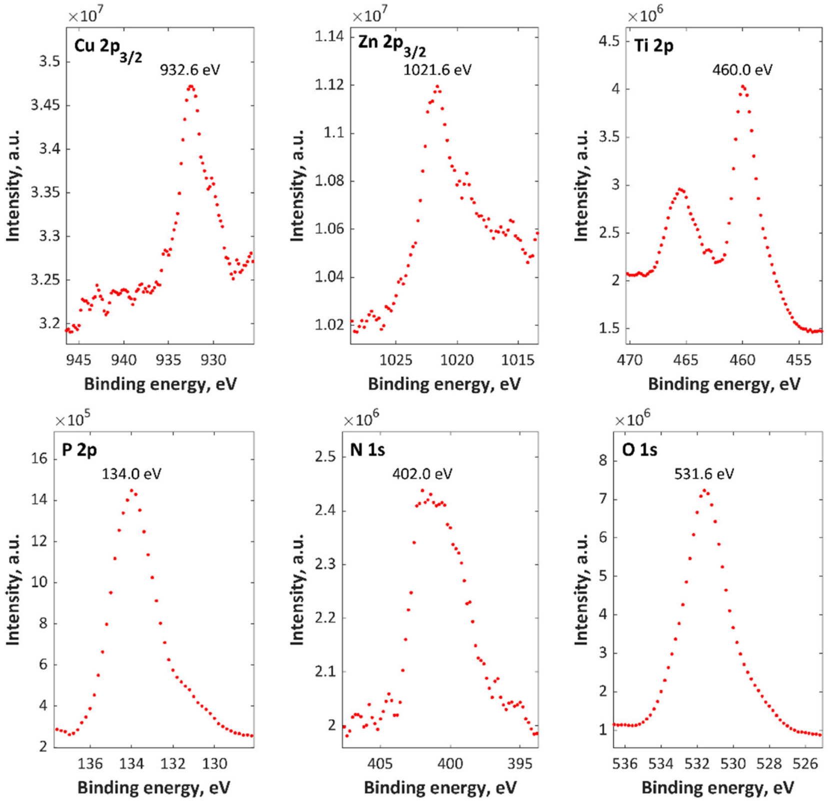

Figure S2. XPS spectra of coatings enriched in zinc and copper, obtained at applied voltage of +400|−35 V.

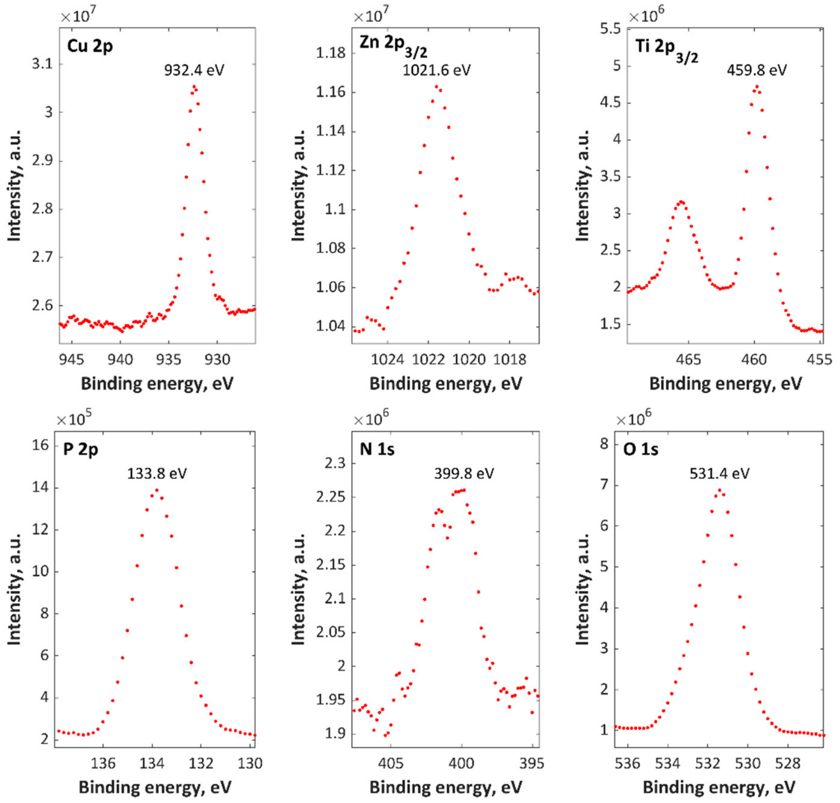

Figure S3. XPS spectra of coatings enriched in zinc and copper, obtained at applied voltage of +400|−135 V.

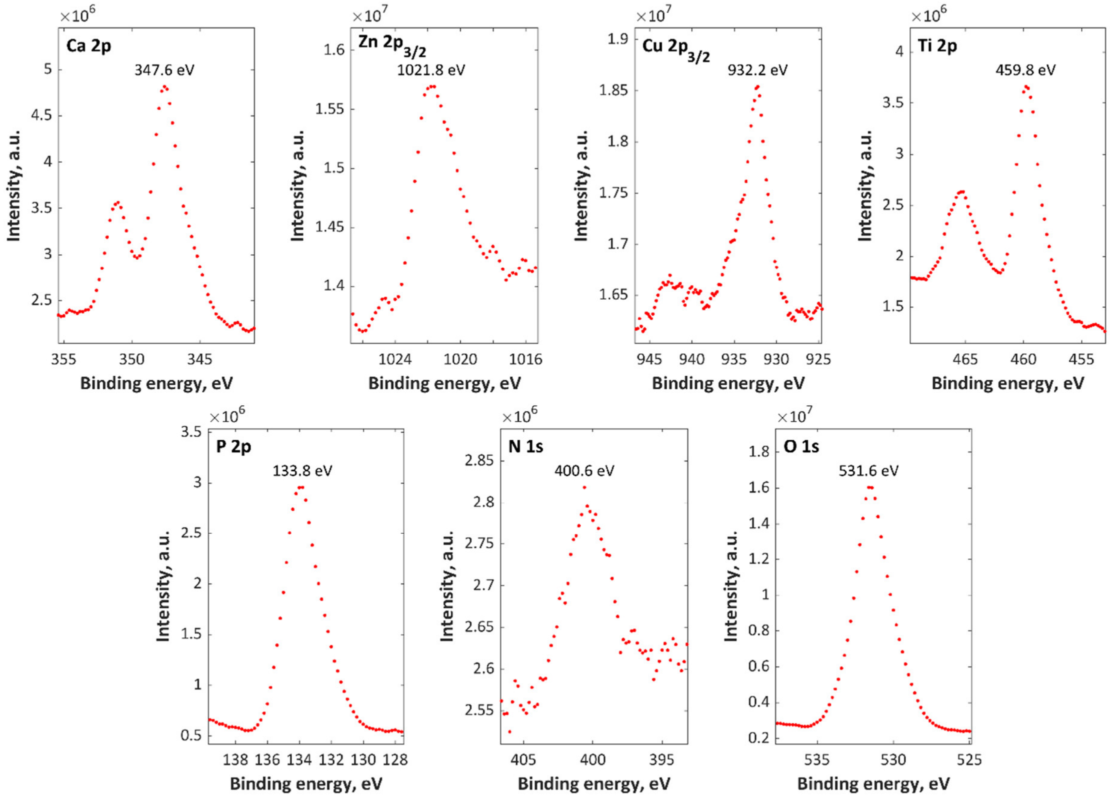

Figure S4. XPS spectra of coatings enriched in zinc, copper, and calcium, obtained at voltage of +400|−35 V.

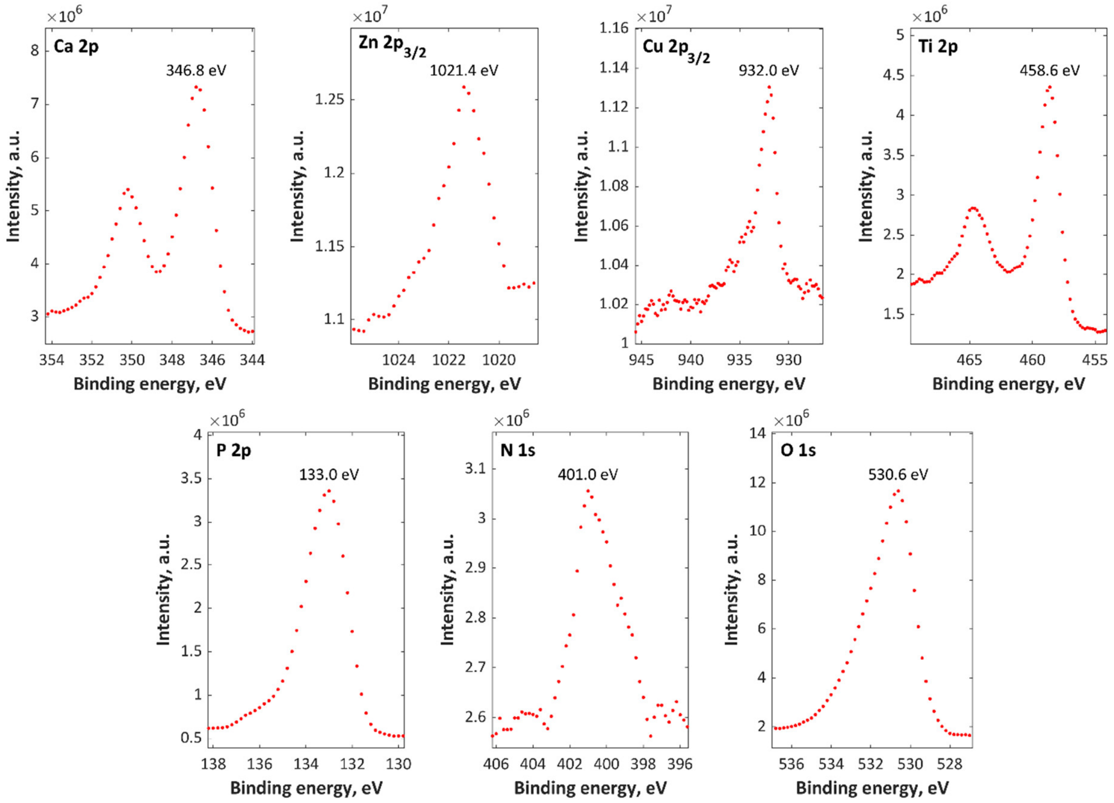

Figure S5. XPS spectra of coatings enriched in zinc, copper, and calcium, obtained at voltage of +400|−135 V.

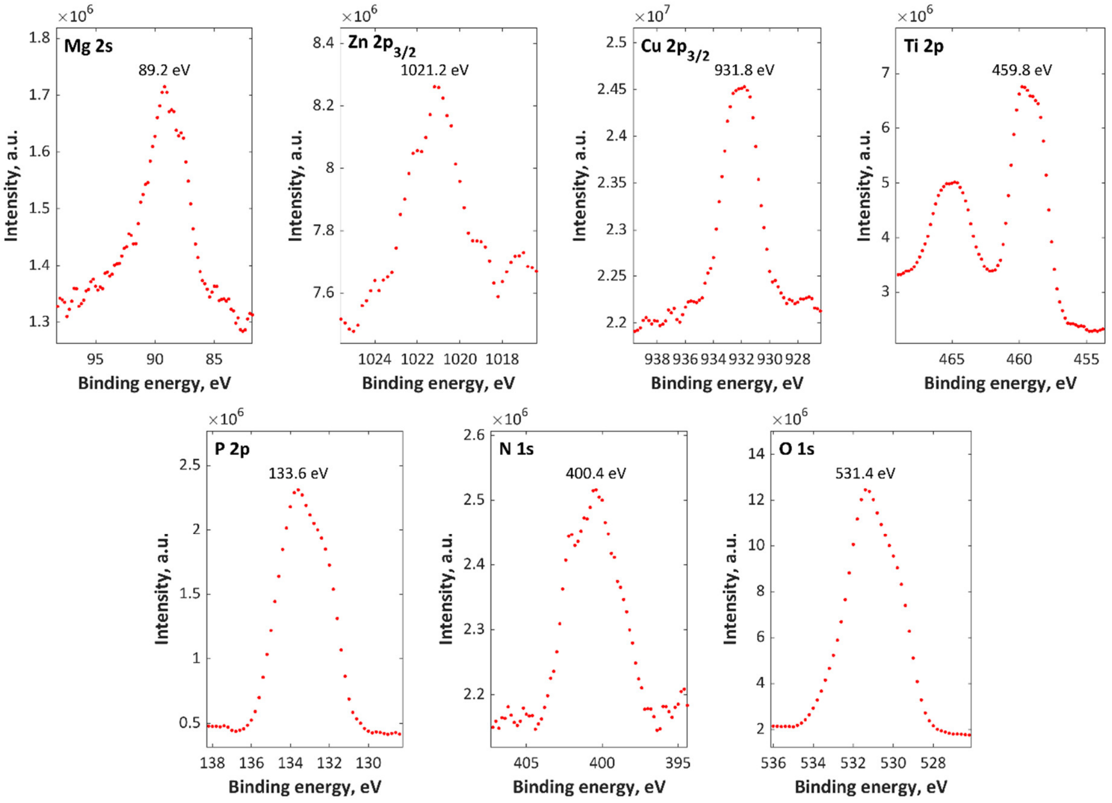

Figure S6. XPS spectra of coatings enriched in zinc, copper, and magnesium, obtained at voltage of +400|–35 V.

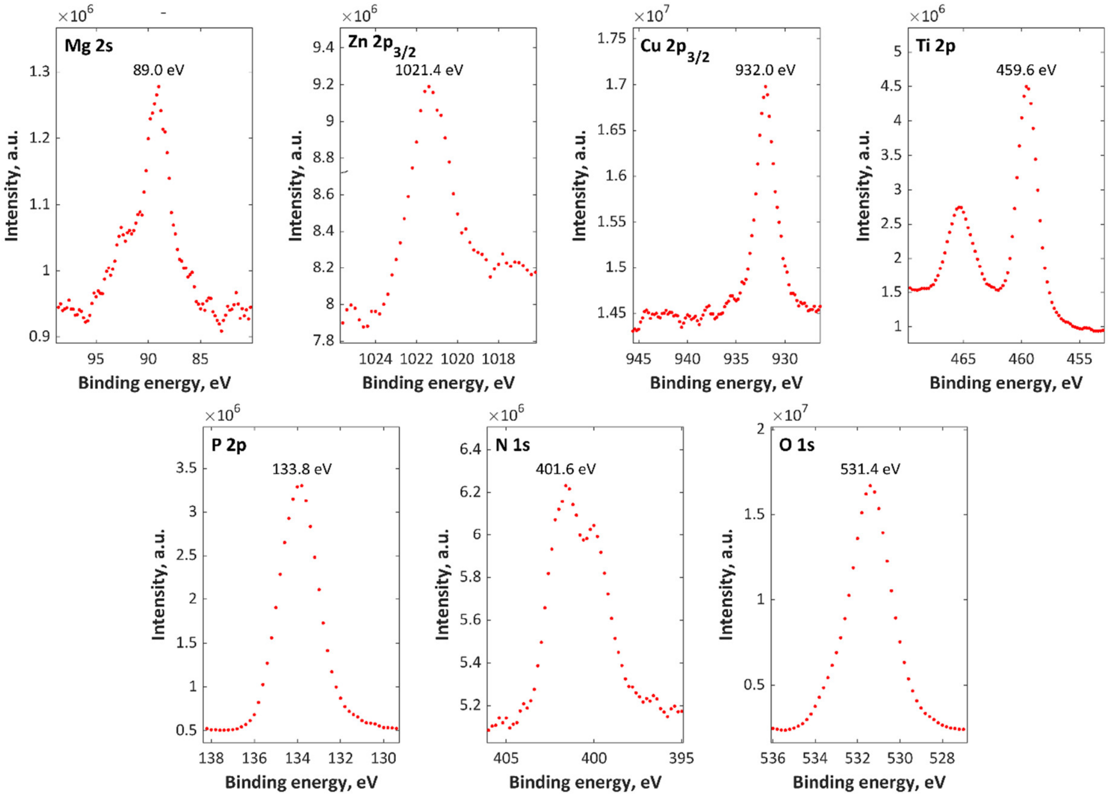

Figure S7. XPS spectra of coatings enriched in zinc, copper, and magnesium, obtained at voltage of +400∣−135 V.

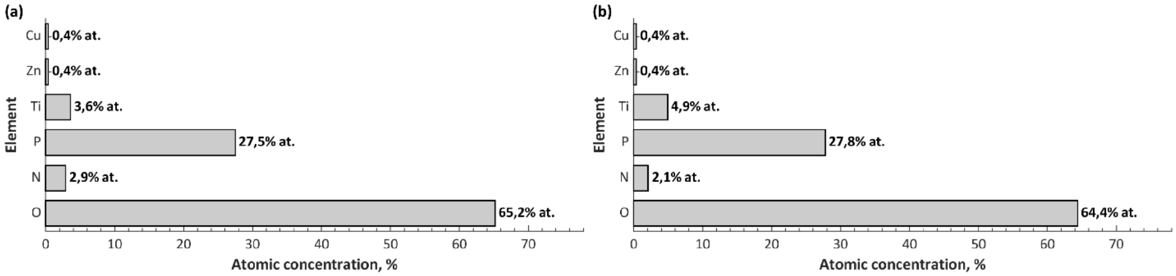

Figure S8. Elemental composition by XPS of top 10 nm of coatings enriched in copper and zinc, obtained at voltage of: (**a**) +400∣−35 V, (**b**) +400∣−135 V.

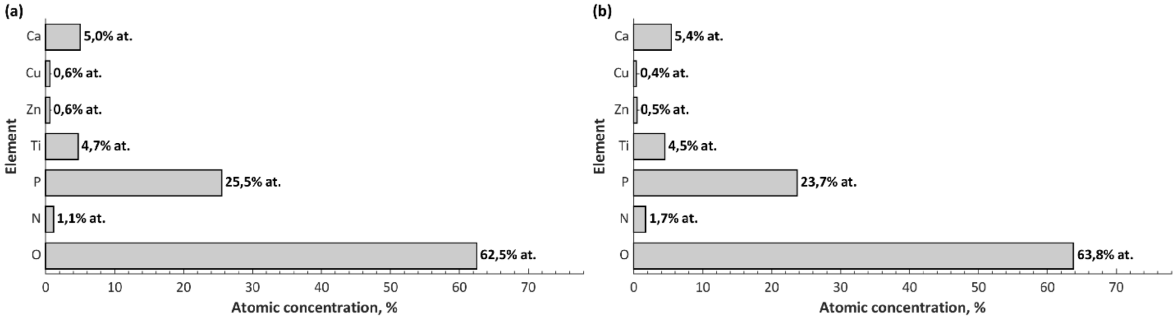

Figure S9. Elemental compositions by XPS of top 10 nm of coatings enriched in copper, zinc, and calcium, obtained at voltages of: (**a**) +400∣−35 V, (**b**) +400 ∣ −135 V.

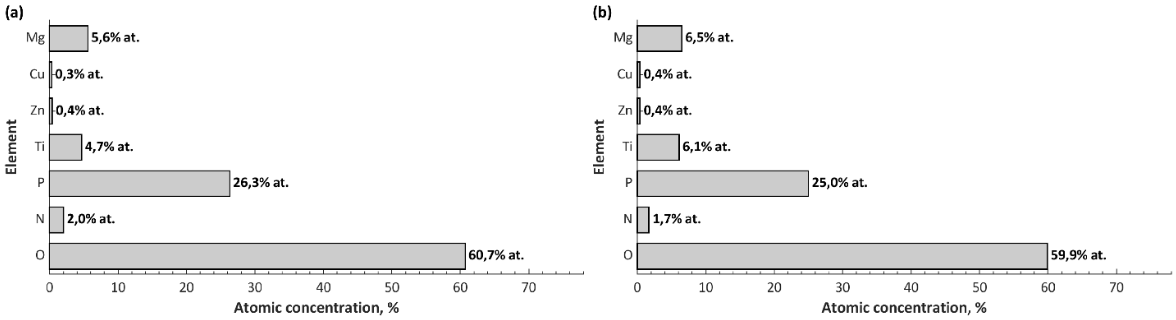

Figure S10. Elemental composition by XPS of top 10 nm of coatings enriched in copper, zinc, and magnesium, obtained at voltages of: (**a**) +400∣−35 V, (**b**) +400∣−135 V.

Table S3. Binding energies (BE, eV) of selected chemical compounds from available literature.

Compounds	BE, eV	Peak	Ref.
**CaO**	**529.4–531.3**	**O 1s**	**[1]**
**Cu_2O**	**530.3**	**O 1s**	**[1]**
**CuO**	**529.6**	**O 1s**	**[1]**
**CuO**	**529.5**	**O1s**	**[3]**
**TiO_2**	**529.9**	**O1s**	**[1]**
**MgO**	**530.0–532.1**	**O 1s**	**[1]**
**Mn_3O_4**	**529.6**	**O 1s**	**[1]**
**$Cu_3(PO_4)_2$**	**531.8**	**O1s**	**[5]**
**$Cu(NO_3)_2$**	**533.4**	**O1s**	**[5]**

$Mg(OH)_2$	530.9	O1s	[1]
$Ca(NO_3)_2$	533.6	O 1s	[1]
$CaHPO_4$	531.7	O 1s	[6]
$Ca_5(PO_4)_3OH$	531.1	O1s	[7]
$Mg_3(PO_4)_2$	532,1	O 1s	[8]
$Zn_3(PO_4)_2$	532.3	O 1s	[9]
P_2O_5	532.2–534.3	O 1s	[1]
Ti_2O_3	457.8	$Ti2p_{3/2}$	[2]
TiO	455.1–455.9	$Ti2p_{3/2}$	[2]
$TiO_{1.5}$	455.2–456.8	$Ti2p_{3/2}$	[2]
$TiO_{0.73}$	454.5	$Ti2p_{3/2}$	[2]
$TiO_{0.9}$	454.7	$Ti2p_{3/2}$	[2]
TiP	454.8	$Ti2p_{3/2}$	[2]
$CaTiO_3$	458.9	$Ti2p_{3/2}$	[2]
$Ti_3(PO_4)_4$	458.8	$Ti2p_{3/2}$	[2]
TiO_2	458.6	$Ti2p_{3/2}$	[2]
CaO	346.1–347.3	Ca $2p_{3/2}$	[1]
$Ca_3(PO_4)_2$	347.7	Ca $2p_{3/2}$	[2]
$Ca(H_2PO_4)_2$	347.6–347.8	Ca $2p_{3/2}$	[2]
$CaHPO_4$	347.5–347.8	Ca $2p_{3/2}$	[2]
$CaHPO_4$	347.4	Ca $2p_{3/2}$	[6]
$Ca_2P_2O_7$	347.6	Ca $2p_{3/2}$	[2]
$Ca(HCOO)_2$	347.4	Ca $2p_{3/2}$	[2]
$Ca(NO_3)_2$	348.7	Ca $2p_{3/2}$	[1]
$Ca_5(PO_4)_3OH$	347.1	Ca $2p_{3/2}$	[7]
MgO	87.9–88.1	Mg 2s	[1]
$Mg_3(PO_4)_2$	90.21	Mg 2s	[8]
ZnO	1021.40–1021.80	Zn $2p_{3/2}$	[2]
$Zn(OH)_2$	1021.80	Zn $2p_{3/2}$	[2]
$Zn_3(PO_4)_2$	1023.3	$Zn3p_{3/2}$	[9]
Zn_3P_2	1020.6	Zn $2p_{3/2}$	[1]
ZnP_2	1020.9	Zn $2p_{3/2}$	[1]
Cu_3P	129.6	P 2p	[1]
CuP_2	129.7	P 2p	[1]
Zn_3P_2	128.3	P 2p	[1]
ZnP_2	129.8	P 2p	[1]
$Mg_3(PO_4)_2$	134.4	P 2p	[8]
$CaHPO_4$	133.6	P 2p	[6]
P_4O_{10}	135.3	P 2p	[1]
P_2O_5	135.2	P 2p	[10]
Cu_2O	932.7	$Cu2p_{3/2}$	[5]
CuO	933.6	$Cu2p_{3/2}$	[5]
$Cu(OH)_2$	934.0	$Cu2p_{3/2}$	[5]
$Cu_3(PO_4)_2$	935.9	$Cu2p_{3/2}$	[5]
$Cu(NO_3)_2$	935.5	$Cu2p_{3/2}$	[5]
$Zn_3(PO_4)_2$	133.4	P 2p	[9]
$Ca_{10}(PO_4)_6(OH)_2$	133.8	P 2p	[2]
$CaHPO_4$	133.8	P 2p	[2]
$Ca_2P_2O_7$	133.8	P 2p	[2]
$Ca(H_2PO_4)_2$	134.0	P 2p	[2]

**$(P_2O_5)_{33}(ZnO)_{67}$**	**134.0**	**P 2p**	**[2]**
**$Ca(H_2PO_4)_2$**	**134.0**	**P 2p**	**[2]**
**$Ca_5(PO_4)_3OH$**	**133.7**	**P $2p_{3/2}$**	**[7]**
**$Cu_3(PO_4)_2$**	**133.9**	**P $2p_{3/2}$**	**[5]**

References:

1. Moulder, J.F.; Stickle,W.F.; Sobol, P.E.; Bomben, K.D. *Handbook of X-ray Photoelectron Spectroscopy*; Chastain, J., Ed.; Perkin-Elmer Corporation: Eden Prairie,MN, USA, **1992**.
2. Wagner, C.D.; Naumkin, A.V.; Kraut-Vass, A.; Allison, J.W.; Powell, C.J.; Rumble, J.R. *NIST Standard*
3. *Reference Database 20*, Version 4.1. **2003.** Available online: http://srdata.nist.gov/xps (accessed on
4. 24 July 2020).
5. Vasquez, R.P. CuO by XPS. *Surface Science Spectra* **1998**, *5(262)*, 261-266, doi: 10.1116/1.1247882.
6. Khairallah, F.; and Glisenti, A. XPS Study of MgO Nanopowders Obtained by Different Preparation Procedures. *Surface Science Spectra* **2006**, *13(58)*, 57-71, doi: 10.1116/11.20060601.
7. Biesinger, M.C. Advanced analysis of copper X-ray photoelectron spectra. *Surface and Interface Analysis* **2017**, *49(9)*, 1325–1334, doi.org/10.1002/sia.6239.
8. Chusuei, C.C.; Goodman, D.W. Brushite (CaHPO4·2H2O) by XPS. *Surface Science Spectra* **2001**, *8(39)*, 38-44, doi: 10.1116/11.20010501.
9. Eighmy, T.T.; Kinner, A.E.; Shaw, E.L.; Eusden, J.D.; Francis, C.A. Hydroxylapatite (Ca5(PO4)3OH) Characterization by XPS: An Environmentally Important Secondary Mineral. *Surface Science Spectra* **1999**, *6(193)*, 192-201, doi: 10.1116/1.1247925.
10. Felker, D.L.; Sherwood, P.M.A. Magnesium Phosphate by XPS. *Surface Science Spectra* **2002**, *9(83)*, 82-90, doi: 10.1116/11.20030104.
11. Felker, D.L.; Sherwood, P.M.A. Zinc Phosphate (Zn3(PO4)2) by XPS. *Surface Science Spectra* **2002**, *9(106)*, 105-113, doi: 10.1116/11.20030107.
12. Wang, Y.; Sherwood, P.M.A. Phosphorus Pentoxide (P2O5) by XPS. urface Science Spectra 2002, 9(159), 158-165, doi: 10.1116/11.20030115.

© 2020 by the authors. Submitted for possible open access publication under the terms and conditions of the Creative Commons Attribution (CC BY) license (http://creativecommons.org/licenses/by/4.0/).
